# Accuracy of bedside ultrasound for predicting resting energy expenditure in critically ill patients: A feasibility study

**DOI:** 10.1371/journal.pone.0325751

**Published:** 2025-06-18

**Authors:** Ming Gao, Li Tan, Yingli Zhou, Wei Peng, Yuan Xu, Hua Zhou, Arthur Raymond Huber van Zanten, Yan Zhu

**Affiliations:** 1 Department of Critical Care Medicine, Beijing Tsinghua Changgung Hospital, School of Clinical Medicine, Tsinghua Medicine, Tsinghua University, Beijing, China; 2 Department of Intensive Care Medicine, Gelderse Vallei Hospital, Ede, The Netherlands; 3 Wageningen University & Research, Wageningen, The Netherlands; University of Alabama at Birmingham, UNITED STATES OF AMERICA

## Abstract

**Objectives:**

This study aimed to assess the accuracy of bedside ultrasound in predicting resting energy expenditure (REE) in critically ill patients.

**Methods:**

We studied critically ill patients admitted to our hospital’s ICU between November 2021 and March 2023 who underwent REE, cardiac ultrasound, and muscle ultrasound evaluations. General demographic information and ultrasound data (including cardiac output, biceps brachii and quadriceps femoris thickness) were collected to estimate REE (REE-US). Simultaneously, REE was measured using indirect calorimetry (REE-IC). Correlations between REE-US and established equations (Harris-Benedict, Penn State University (PSU), Mifflin, ASPEN standard) as well as REE-IC were evaluated. Additionally, the feasibility and application of ultrasound for REE prediction across different disease conditions in critically ill patients were analysed.

**Results:**

Ninety-seven critically ill patients with 124 ultrasound measurements were included. The Penn State University formula showed the highest correlation with REE-IC (r = 0.779, p < 0.001), followed by ultrasound estimation (r = 0.668, p < 0.001). Correlation between the PSU formula and REE-IC remained robust across subgroups. However, REE-US correlation was weaker in patients with low BMI (BMI < 20 kg/m^2^) (r = 0.521, p = 0.009) but comparable to the PSU formula in normal and high BMI groups (BMI 20–30 kg/m^2^: r = 0.682 vs. r = 0.714, p = 0.5743; BMI > 30 kg/m^2^: r = 0.712 vs. r = 0.882, p = 0.1294). In subgroup analysis, REE-US performed similarly to the PSU formula in the sepsis subgroup (r = 0.612 vs r = 0.661, p = 0.6852) and ICU patients in the late period of the acute phase (r = 0.675 vs r = 0.751, p = 0.2762).

**Conclusions:**

The Penn State University formula demonstrated the strongest correlation with REE-IC in critically ill patients. Ultrasound may replace the PSU formula in non-mechanically ventilated patients with unavailable gas measurement parameters. However, ultrasound-derived REE is less predictive in patients with low BMI or during the early acute phase of critical illness. Further research is warranted to refine ultrasound application in these populations.

## Introduction

Nutritional optimisation tailored to individual metabolic states is critical for improving the efficacy and safety of nutritional interventions in critically ill patients. Extensive observational studies and guidelines have underscored the non-linear relationship between energy provision and mortality among this patient population, highlighting the detrimental effects of persistent underfeeding and overfeeding [1,2]. Consequently, precise and real-time assessment of energy requirements is paramount.

Resting energy expenditure (REE), primarily driven by lean body mass metabolism, is influenced by weight, height, sex, and age. While predictive equations offer reliability in estimating REE among healthy individuals, critically ill patients exhibit dynamic variations in REE across different disease stages and among individuals, owing to stress-related factors. Indirect calorimetry (IC), employing measurements of oxygen consumption (VO_2_) and carbon dioxide production (VCO_2_), serves as the gold standard for REE evaluation [3,4]. However, its clinical utility is hampered by equipment limitations, technical demands, and operator expertise.

In recent years, bedside ultrasound has become increasingly common in the field of critical care medicine, and its reliability in cardiac function assessment, muscle measurement, lung assessment, and other areas has been widely recognized. In addition, due to its fast access, bedside operation, and non-invasive features, bedside ultrasound has gradually become a prevalent choice in ICU, playing an important role in directing the treatment of critically ill patients and improving prognosis [5–7]. Emerging evidence suggests the feasibility of estimating REE through ultrasound-based formulas, utilising indicators like cardiac output (CO) and muscle thickness assessed via bedside ultrasound in critical care settings [8]. This preliminary study has demonstrated a promising correlation between ultrasound-based methods and IC. Therefore, this study aims to retrospectively assess the predictive efficacy of ultrasound and other formulaic approaches for evaluating energy metabolism in critically ill patients, focusing on elucidating the patient populations most suitable for ultrasound-based assessments.

## Materials and methods

The study was approved by the Ethics Committee of Beijing Tsinghua Changgung Hospital (approval number: 16124−0110). The procedures used in this study were performed in accordance with the ethical standards as laid down in the 1964 Declaration of Helsinki and its later amendments or comparable ethical standards. Informed consent was obtained from all patients and their authorized immediate family members. It included critically ill patients admitted to the Department of Critical Care Medicine between November 2021 and March 2023. Informed consent was obtained from all patients and their authorized immediate family members.

Inclusion Criteria:

1. Age range: 18–80 years.
2. Critically ill patients who underwent monitoring of resting energy expenditure (REE) using the indirect calorimetry (IC) method, cardiac ultrasound, and muscle ultrasound simultaneously during their ICU stay. Cardiac ultrasound recordings must include cardiac output (CO), while muscle ultrasound measurements must encompass biceps brachii thickness of both upper limbs and quadriceps femoris thickness of both lower limbs.
3. The time interval between REE monitoring and ultrasound examination was ≤ 12 hours, with no significant vital sign fluctuations during this period.
4. Expected length of ICU hospitalisation exceeding 48 hours.

Exclusion Criteria:

1. Patients contraindicated for IC method use, such as those requiring a fraction of inspired oxygen > 0.6 or continuous positive airway pressure > 14 cm H2O and those with chest tube air leakage.
2. Patients with poor ultrasonic windows.
3. Patients with limb deficiencies.
4. Pregnant patients.

### Study protocol

Patient demographics, including age, sex, height, weight, ICU diagnosis, ICU admission time, Acute Physiology and Chronic Health Evaluation (APACHE) II score, and modified Nutrition Risk in Critically Ill (mNUTRIC) score were recorded. Resting energy expenditure (REE) was measured using indirect calorimetry (IC) with a COSMED metabolic cart (model: S/N 2019091309) from Italy.

The measurement methods for cardiac output (CO) and muscle layer thickness (MLT) followed protocols described in previous studies [8]. All ultrasound monitoring including CO and MLT were performed by the same one intensive care physician, who had completed Chinese intensive care ultrasound-related training and obtained a certification, and ultrasound quality control was performed by a senior physician, who was a member of critical care ultrasound study group in China. The standard operation process was described in detail in the supplementary file (S1 Appendix in S1 File)

Results of CO and MLT were inputted into the ultrasound formula to calculate the predictive REE value (ultrasound formula: REE-US = 206 + 173.5 × CO (L/min) + 137 × MLT (cm) – 230 × (female = 1; male = 0)).

Other energy prediction formulas were computed as follows:

- Harris-Benedict formula: For males: 66 + 13.75 × body weight (kg) + 5 × height (cm) – 6.8 × age (years); For females: 655 + 9.6 × body weight (kg) + 1.8 × height (cm) – 4.7 × age (years).
- Mifflin St. Jeor formula: For males, 10 × body weight (kg) + 6.25 × height (cm) – 5 × age (years) + 5; for females, 10 × body weight (kg) + 6.25 × height (cm) – 5 × age (years) – 161.
- Penn State University (PSU) formula: REE = 0.96 × Mifflin St. Jeor formula + 167 × body temperature (°C) + 31 × minute ventilation (L/min) – 6212.
- Modified Penn State University (mPSU) formula: REE = 0.71 × Mifflin St. Jeor formula + 85 × body temperature (°C) + 64 × minute ventilation (L/min) – 3085. This formula applies to patients aged ≥ 60 years with a BMI ≥ 30 kg/m^2^.
- ASPEN standard: simplistic weight-based equation 25 kcal/kg/d.

### Statistical analysis

Normally distributed measurement data are presented as mean ± standard deviation (X ± SD), while non-normally distributed data are presented as median (and interquartile ranges (IQR)). Vital signs at two different time points, REE-IC and REE-US, were compared using paired sample test. If the data were normally distributed and homogeneously variant data, paired sample t-test was used. If the data were non-normally distributed or heterogeneously variant data, Wilcoxon signed rank test was used.. Consistency between methods was assessed using the Bland-Altman method, with a deviation value < ±10% indicative of good consistency. Correlation analysis was used to assess the relationship between the IC method and each prediction formula, with calculated correlation coefficients (R values). Statistical analysis was conducted using SPSS 24.0 software. The cocor software [9] was employed to compare differences between correlation coefficients. A significance level set at P < 0.05 indicates statistical significance.

## Results

A total of 97 critically ill patients with 124 ultrasound measurements were included, and their ICU admission reasons are detailed in Table 1. Among them, 64 were males, with a median age of 66.0 (52.5, 74.0) years, a median mNUTRIC score of 5 (3.5, 6.0), and an APACHE-II score of 18.3 ± 8.6 (Table 1). The time interval between IC and ultrasound methods was 224.0 ± 150.1 minutes, with no significant differences in vital signs observed between monitoring time points (Table 2). Among the 97 patients included, 93.8% used sedatives or analgesics at least once throughout their entire ICU stay, and 23.7% used muscle relaxants at least once (Table 1). In 124 REE-IC measurements, a total of 58 measurements were performed while the patient was using sedatives, accounting for 46.8% of the total number of measurements; among the 124 REE-US measurements, a total of 59 measurements were taken while the patient was using sedatives, accounting for 47.6% of the total measurements. The median RASS score for patients using sedatives was −2 (S1 and S2 Tables in S2 Appendix (S1 File)). The proportion of patients using sedatives or analgesics was not significantly different between IC and ultrasound monitoring time points (Table 2).

**Table 1:** Demographic data and baseline characteristics.

Variable	Total population (n = 97)
Age, y (median, IQR)	66.0 (21.5)
Male (n, %)	64, 65.9%
BMI, kg/m^2^ (median, IQR)	22.9 (4.8)
APACHE-II (mean±SD)	18.3 ± 8.6
Respiratory quotient (mean±SD)	0.81 ± 0.10
REE_IC, kcal/d (mean±SD)	1505.6 ± 385.3
CO, L/min (mean±SD)	4.88 ± 1.27
Muscle layer thickness, cm (mean±SD)	4.42 ± 1.12
mNUTRIC score (median, IQR)	5 (2.5)
Admission diagnosis	
Surgical (n, %)	50 (51.5)
Pneumonia (n, %)	20 (20.6)
Other infectious disease (n, %)	11 (11.3)
Hemorrhagic disorder (n, %)	4 (4.1)
Cardiac insufficiency (n, %)	3 (3.1)
Pulmonary embolism (n, %)	2 (2.1)
Other cases^*^ (n, %)	7 (7.2)
Drug use^#^	
Sedatives	
Propofol (n, %)	86 (88.7)
Midazolam (n, %)	47 (48.5)
Dexmedetomidine (n, %)	66 (68.0)
Others (n, %)	5 (5.2)
Analgesics	
Fentanyl (n, %)	86 (88.7)
Remifentanil (n, %)	18 (18.6)
Morphine (n, %)	9 (9.3)
Others (n, %)	13 (13.4)
Muscle relaxants (n, %)	23 (23.7)

BMI: Body Mass Index; APACHE: Acute Physiology and Chronic Health Evaluation; REE: Rest Energy Expenditure; IC: Indirect Calorimetry; CO: Cardiac Output; mNUTRIC: modified Nutrition Risk in the Critically ill, IQR: interquartile range, SD: standard deviation

* Other cases include 1 case of DKA, 1 case of traffic accident injury, 1 case of gastrointestinal perforation, 1 case of pancreatitis, 1 case of blood disorder, 1 case of refeeding syndrome, and 1 case of epilepsy.

# Because patients may switch between different types of sedatives and analgesics during hospitalization, and multimodal analgesia and sedation may be adopted, the total percentage of all medications exceeds 100%.

**Table 2:** Vital signs during indirect calorimetry and ultrasound measurements.

Variable	REE-IC	Ultrasound	P-Value
Body temperature(°C) (median, IQR)	36.8 (0.95)	36.9 (1.075)	0.242
Heart rate(bpm) (median, IQR)	82.0 (23.75)	80.0 (21.0)	0.422
Systolic blood pressure(mmHg) (mean±SD)	130.2 ± 20.4	131.0 ± 19.3	0.485
Diastolic blood pressure(mmHg) (median, IQR)	60.0 (16.75)	59.5 (13.75)	0.083
SpO2(%) (median, IQR)	100.0 (3)	100.0 (2)	0.071
Sedatives (N, %)	58 (46.8%)	59 (47.6%)	0.899
Analgesics (N, %)	95 (76.6%)	94 (75.8%)	0.881

REE: Resting energy expenditure, IC: indirect calorimetry, IQR: interquartile range, SD: standard deviation, SpO2: pulse oximetry oxygen saturation

The REE-IC was 1,505.60 ± 385.25 kcal/day in the entire cohort, with detailed IC monitoring parameters listed in the supplementary file S3 Table in S1 File. REE values predicted by Mifflin, Harris-Benedict, Penn State, and ASPEN standard (25 kcal/kg/day) formulas were 1,356 ± 242 (r = 0.614, p < 0.001), 1,377 ± 253 (r = 0.590, p < 0.001), 1,569 ± 314 (r = 0.779, p < 0.001), and 1,649 ± 343 (r = 0.475, p < 0.001), respectively. The REE-US was 1,589 ± 341 (r = 0.668, p < 0.001). Correlation coefficients indicated the highest agreement between REE-IC and PSU formula, followed by REE-US, while Mifflin and Harris-Benedict formulas demonstrated poorer prediction accuracy (Table 3).

**Table 3:** Correlation coefficients between different formulas and REE-IC for different BMI groups.

		Mifflin formula (kcal/d)	Harris-Benedict formula (kcal/d)	Penn State University formula (kcal/d)	Ultrasound (REE-US) (kcal/d)	ASPEN standard (kcal/d)	REE-IC (kcal/d)
Total measurements (n = 124)		1356 ± 242 r = 0.614, p < 0.001	1377 ± 253 r = 0.590, p < 0.001	1569 ± 314 r = 0.779, p < 0.001	1589 ± 341 r = 0.668, p < 0.001	1649 ± 343 r = 0.475, p < 0.001	1506 ± 385
BMI (kg/m^2^)	BMI < 20 (n = 24)	1164 ± 220 r = 0.842, p < 0.001	1134 ± 190 r = 0.864, p < 0.001	1359 ± 307 r = 0.872, p < 0.001	1470 ± 255 r = 0.521, p = 0.009	1248 ± 163 r = 0.771, p < 0.001	1341 ± 402
	BMI 20–30 (n = 88)	1379 ± 205 r = 0.494, p < 0.001	1403 ± 200 r = 0.471, p < 0.001	1602 ± 258 r = 0.714, p < 0.001	1602 ± 338 r = 0.682, p < 0.001	1680 ± 237 r = 0.435, p < 0.001	1536 ± 361
	BMI > 30 (n = 12)	1569 ± 293 r = 0.707, p = 0.010	1678 ± 297 r = 0.770, p = 0.003	1739 ± 360 r = 0.882, p < 0.001	1734 ± 448 r = 0.712, p = 0.009	2226 ± 311 r = 0.582, p = 0.047	1615 ± 459

Note: 97 critically ill patients with 124 ultrasound measurements were included. N represents the number of measurements instead of the number of patients.

r is the correlation coefficient between different prediction formulas and REE measured by the IC method.

Abbreviations: ASPEN: American Society for Parenteral and Enteral Nutrition; REE-IC: resting energy expenditure using indirect calorimetry; REE-US: resting energy expenditure using ultrasound; Kcal/d: kilocalorie per day; BMI: Body Mass Index; r: correlation coefficient.

### Subgroup analyses

To assess formula performance, BMI stratified 124 ultrasound measurements into three groups (BMI < 20 kg/m^2^, BMI 20−30 kg/m^2^, and BMI > 30 kg/m^2^). High correlation coefficients between the PSU formula and REE-IC were observed across all groups. However, for patients with BMI < 20 kg/m^2^, REE-US correlation with REE-IC was suboptimal (1,470 ± 255 vs 1,341 ± 402, r = 0.521, p = 0.009), even lower than Mifflin and Harris-Benedict formulas (Table 3). Acceptable correlations were found for normal and high BMI groups, with REE-US demonstrating superiority over Mifflin and Harris-Benedict formulas, comparable to the PSU formula in the BMI 20−30 kg/m^2^ group (r = 0.682 vs r = 0.714, p = 0.5743, 95% CI: −0.1554 to 0.2803). REE-US also showed acceptable predictive value for obese patients (BMI > 30 kg/m^2^) (Table 5).

**Table 4:** Correlation coefficients between different formulas and REE-IC in different phases of ICU stay.

		Mifflin formula (kcal/d)	Harris-Benedict formula (kcal/d)	Penn State University formula (kcal/d)	Ultrasound (REE-US) (kcal/d)	ASPEN standard (kcal/d)	REE-IC (kcal/d)
Total measurements (n = 124)		1356 ± 242 r = 0.614, p < 0.001	1377 ± 253 r = 0.590, p < 0.001	1569 ± 314 r = 0.779, p < 0.001	1589 ± 341 r = 0.668, p < 0.001	1649 ± 343 r = 0.475, p < 0.001	1506 ± 385
Different stages	Early period of acute phase (n = 61)	1333 ± 269 r = 0.664, p < 0.001	1372 ± 264 r = 0.607, p < 0.001	1549 ± 356 r = 0.800, p < 0.001	1581 ± 356 r = 0.668, p < 0.001	1628 ± 358 r = 0.528, p < 0.001	1509 ± 436
	Late period of acute phase (n = 63)	1378 ± 212 r = 0.576, p < 0.001	1382 ± 243 r = 0.573, p < 0.001	1588 ± 270 r = 0.751, p < 0.001	1597 ± 328 r = 0.675, p < 0.001	1669 ± 330 r = 0.411, p = 0.001	1503 ± 333

Note: 97 critically ill patients with 124 ultrasound measurements were included. N represents the number of measurements instead of the number of patients.

Early period of acute phase: ICU admission time ≤ 3 days. Late period of acute phase: ICU admission time > 3 days.

r is the correlation coefficient between different prediction formulas and REE measured by the IC method.

Abbreviations: ASPEN: American Society for Parenteral and Enteral Nutrition; REE-IC: resting energy expenditure using indirect calorimetry; REE-US: resting energy expenditure using ultrasound; Kcal/d: kilocalorie per day; BMI: Body Mass Index; r: correlation coefficient.

**Table 5:** Comparison of correlation coefficients of the ultrasound method and the Penn State University equation in different populations to estimate resting energy expenditure.

		Correlation coefficient between the Penn State University equation and Indirect Calorimetry (REE)	Correlation coefficient between the ultrasound method and Indirect Calorimetry (REE)	Comparison of correlation coefficients of the ultrasound method and the Penn State University equation P value^*^, (95% confidence interval)
Total measurements (n = 124)		R1 = 0.779	R2 = 0.668	P = 0.0124, (0.0509 to 0.4205)
BMI	BMI < 20 (n = 24)	R1 = 0.872	R2 = 0.521	P = 0.0024, (0.2716 to 1.2558)
	BMI 20–30 (n = 88)	R1 = 0.714	R2 = 0.682	P = 0.5743, (−0.1554 to 0.2803)
	BMI > 30 (n = 12)	R1 = 0.882	R2 = 0.712	P = 0.1294, (−0.1443 to 1.1313)
Measurements with sepsis (n = 33)		R1 = 0.661	R2 = 0.612	P = 0.6852, (−0.3163 to 0.4812)
Different stages	Early period of acute phase (n = 61)	R1 = 0.800	R2 = 0.668	P = 0.0228, (0.0406 to 0.5423)
	Late period of acute phase (n = 63)	R1 = 0.751	R2 = 0.675	P = 0.2762, (−0.1243 to 0.4350)

Note: 97 critically ill patients with 124 ultrasound measurements were included. N was represented the number of measurements instead of the number of patients.

R1 is the correlation coefficient between Penn State University equation and REE measured by Indirect Calorimetry (REE)

R2 is the correlation coefficient between ultrasound method and REE measured by Indirect Calorimetry (REE).

* P value is the comparison of the difference between coefficients R1 and R2

Furthermore, among the 124 measurements, 33 were measured when patients had sepsis, and the Mifflin and Harris-Benedict formulas exhibited low correlation coefficients with REE-IC. The PSU formula and REE-US showed similar correlations, with no statistical difference observed (r = 0.612 vs r = 0.661, p = 0.6852, 95% CI: −0.3163 to 0.4812) (S4 Table in supplementary file S4 Appendix (S1 File), and Table 5).

Finally, a comparison of different formula predictive values for REE in the early (ICU admission time ≤ 3 days) versus the late period of the acute phase (ICU admission time > 3 days) revealed statistically significant differences between REE-US and Penn State formula in the early disease course. However, no significant differences were observed in the late period of the acute phase (r = 0.675 vs r = 0.751, p = 0.2762, 95% CI: −0.1243 to 0.4350) (Tables 4 and 5).

## Discussion

Measuring resting energy expenditure via indirect calorimetry facilitates personalised nutritional interventions in critically ill patients, contributing to improved clinical outcomes such as lower infection rates and reduced hospitalisation durations [10,11]. However, equipment and personnel constraints hinder the clinical application of IC. The increasing use of ultrasound in the ICU offers a non-invasive and easily accessible alternative for monitoring cardiac and muscle functions [12–14]. By assessing cardiac output and muscle thickness, this study evaluated the correlation between ultrasound-derived REE (REE-US) and REE measured by IC, assessing its clinical feasibility and applicability across different patient populations [8].

At present, there are many energy prediction formulas in clinical practice. This study selected three common formulas in ICU and the recommended kilogram weight method (25 kcal/kg/d) in guidelines for comparison. It can be seen that the indicators included in different prediction formulas are not the same. The Harris-Benedict formula was developed in 1919, while the Mifflin formula was developed in 1990, either of which was developed based on a healthy population. The Harris-Benedict formula was similar to the Mifflin equation in the form and intent, which was used to predict resting energy expenditure based on anthropometrics (weight, height, age and gender). However, disease-related indicators were not included for the two formulas above. Therefore, it is not surprising that these formulas did not work well in critically ill patients. Besides, ASPEN (American Society for Parenteral and Enteral Nutrition) recommended that a predicted value of 25 kcal/kg/d can be used for energy supply in patients who cannot undergo energy metabolism monitoring. However, it can be seen that this recommendation is only related to the patient’s weight and has significant bias in clinical use. Penn State University formula was developed based on a mixed ICU population of 169 participants in Pennsylvania, which not only incorporated relevant indicators of the Mifflin St. Jeor formula, but also included body temperature and minute ventilation, which are often related to disease status, oxygen supply, and oxygen consumption.

The Penn State University formula, incorporating parameters specific to critical illness metabolism, has demonstrated superior accuracy to traditional formulas such as the Harris-Benedict formula [15,16]. Consistent with previous findings, our study observed a strong correlation between the Penn State formula and REE-IC, particularly in obese patients (BMI > 30 kg/m^2^) [17]. Nonetheless, limitations exist in the clinical application of PSU, especially in non-mechanically ventilated patients, where specific parameters like minute ventilation are unavailable.

Recent studies have shown promising results for REE estimation using ultrasound, providing a feasible alternative when gas measurements are inaccessible [8]. Our findings suggest that ultrasound-derived REE performs comparably to the Penn State formula in patients with BMI ≥ 20 kg/m^2^, sepsis, and stable conditions.

Our study found that there was no statistical difference between the correlation coefficient between ultrasound and the Penn State formula in patients with BMI ≥ 20 kg/m^2^, sepsis patients and patients with middle and late disease courses (with stable condition), suggesting that when gas measurement parameters such as VO_2_, VCO_2_, and minute ventilation are not available in patients without mechanical ventilation, the ultrasound method can also evaluate resting energy expenditure to a certain extent.

Muscle thickness measured by ultrasound offers a simple and rapid assessment, with results demonstrating a high correlation with computed tomography despite the potential influence of fluid balance changes in critically ill patients [18–21]. It should be noted that for both weight and muscle thickness, the change may be related to the positive fluid balance frequently encountered among critically ill patients, especially during early fluid resuscitation. This may also explain why the REE-US method showed poorer predictive performance in the acute phase. However, patients are often intubated during the acute phase, allowing REE monitoring via IC. However, accessing IC may be challenging upon extubation or in patients requiring non-invasive ventilation (NIV). Ultrasound then presents an advantage in these scenarios, particularly during the stable phase of illness, where it exhibits higher predictive performance. Despite its advantages, caution is warranted regarding the application of REE-US in specific populations.

Previous studies have shown that predictive equations for REE are based on anthropometrics, e.g., BMI, fat-free mass, age, sex, disease-related conditions, and combinations of these factors. Extremes of weight affect prediction errors significantly [22,23]. Specifically, our study indicated that REE-US showed poor predictive value in patients with extremely low BMI (< 20 kg/m^2^), necessitating careful formula selection for this population. It is unclear why REE-US has poor prediction in critically ill individuals with low BMI. On the one hand, extremely low BMI may indicate that the patient’s disease severity is more severe and the duration of the disease is longer. In these patients, REE may be associated not only with indicators such as CO and muscle thickness, but also with other indicators such as the severity of inflammation and the quality of muscle mass, rather than just muscle weight or thickness. Recent studies have suggested that the resting metabolic rate of patients with different BMI is believed to be correlated with lean body mass, body weight, and extracellular fluid [24]. Therefore, further inclusion of some body composition measurement indicators in patients with low BMI may help improve the predictive ability of the formula.

Additionally, metabolic variations between acute and stable disease stages may impact formula applicability [25–27]. Our study observed a suboptimal correlation between ultrasound-derived predictions and REE during the acute phase (≤ 3 days), likely attributed to infection severity and fluid overload affecting CO and muscle ultrasound accuracy. Conversely, in stable disease stages, ultrasound-derived predictions showed acceptable performance, comparable to the PSU formula.

When comparing our study with the original investigation on REE-US, discrepancies in correlation coefficients between ultrasound and IC methods were evident, likely attributable to population differences. Specifically, our study, conducted among a Chinese population, revealed lower cardiac output (CO) values compared to the original cohorts (4.88 ± 1.27 L/min versus 7.3 ± 1.8 and 9.2 ± 1.9 L/min, respectively) [8]. Furthermore, our cohort exhibited a lower mean BMI (26.8 ± 4.5 versus 27.7 ± 4.6), along with older age and higher APACHE-II scores, underscoring the imperative for additional validation, particularly in elderly and critically ill patients.

### Limitations

Firstly, this study was retrospective, encompassing patients who underwent both ultrasound examination and IC monitoring. As the examinations were independent, their order was not specified. Nonetheless, the average interval between them was approximately 4 hours (224.0 ± 150.1 min), with no discernible vital sign fluctuations (Table 2). This may suggest relative stability and comparability in the patient’s metabolic status. In addition, although this study is a retrospective study, the quality of ultrasound and the results are relatively reliable. On the one hand, intensive care ultrasound technology in our ICU has been relatively mature, and it was done by a fixed intensive care physicians, on the other hand, our institution has an established quality assurance process where images are reviewed shortly after captured by a separate senior physician. Secondly, while we expanded the sample size to evaluate the ultrasound formula’s predictive value across different populations based on previous research, it remains a retrospective study with a modest sample size. Further validation through prospective controlled studies is warranted. Thirdly, patients were not monitored after 5 hours of fasting by the energy metabolism cart in the morning, but rather when their condition stabilised (stable vital signs, no physical activity, or other stimuli). Although the optimal REE measurement time typically involves fasting for over 5 hours, this condition is rarely met in critically ill patients, who often receive continuous enteral or parenteral nutrition. Some studies propose that REE can still be measured in critical care settings, even during continuous feeding, as the change in metabolic rate is negligible [28]. Thus, a minimum 5-hour fasting period may not be necessary during REE monitoring. While this may slightly affect REE accuracy, the short interval between ultrasound and REE minimises this effect. Fourthly, sedatives, analgesics, and even muscle relaxants are relatively common in our ICU, which is consistent with the severity of the patient’s condition. In theory, the use of these drugs may affect the REE levels of patients, especially during deep sedation [29,30]. However, some studies have found that the relationship between sedatives and REE is not clear [31,32], which may be related to the patient’s primary disease and overall condition. On the one hand, we believe that the resting metabolic rate of patients may decrease during deep sedation, but the effect of shallow sedation on REE may not be significant. In this population, the median RASS score was −2, indicating a mild sedation state and possibly less affected by sedative drugs. On the other hand, there was no significant difference in the proportion of sedative and analgesic drugs used at the two time points of REE-US and REE-IC, which eliminated the effect of sedative and analgesic drugs on this study to some extent. In addition, the main purpose of this study was to verify the accuracy of the previous REE-US formula, so no other parameters were added to the existing formula. Of course, this also proposes new ideas for further optimizing ultrasound formula parameters in the future. Finally, while the correlation coefficient between the ultrasound prediction formula and the gold standard was favourable in some populations, substantial absolute value differences persisted (S1–S24 Figs in S5 Appendix (S1 File)), mirroring the original study. Excessive disparities in predicted values can lead to inadequate or excessive energy supply in clinical settings. Therefore, further optimisation of formula parameters in a larger population is necessary, potentially requiring different coefficients for different populations to refine ultrasound formula results.

## Conclusions

The Penn State University formula demonstrates the highest correlation with IC-measured REE in critically ill patients. Ultrasound-derived REE estimation holds promise as an alternative in select patient populations lacking mechanical ventilation. However, caution is warranted in patients with low body weight and during acute disease stages. Further validation and formula optimisation are necessary for widespread clinical adoption.

## Supporting information

S1 File S1 Table. Sedatives and analgesics use during REE-IC monitoring. S2 Table. Sedatives and analgesics use during REE-US monitoring. S3 Table. Oxygen therapy parameters and other parameters of patients before REE monitoring. S4 Table. Correlation coefficients between different formulas and REE-IC in sepsis. S1 Fig. Comparison of REE-IC and REE-US in patients with BMI < 20 kg/m2. S2 Fig. Comparison of REE-IC and REE-Penn State University in patients with BMI < 20 kg/m2. S3 Fig. Comparison of REE-IC and REE-Harris-Benedict in patients with BMI < 20 kg/m2. S4 Fig. Comparison of REE-IC and REE-Mifflin in patients with BMI < 20 kg/m2. S5 Fig. Comparison of REE-IC and REE-US in patients with BMI 20–30 kg/m2. S6 Fig. Comparison of REE-IC and REE-Penn State in patients with BMI 20–30 kg/m2. S7 Fig. Comparison of REE-IC and REE-Harris-Benedict in patients with BMI 20–30 kg/m2. S8 Fig. Comparison of REE-IC and REE-Mifflin in patients with BMI 20–30 kg/m2. S9 Fig. Comparison of REE-IC and REE-US in patients with BMI > 30 kg/m2. S10 Fig. Comparison of REE-IC and REE-Penn State in patients with BMI > 30 kg/m2. S11 Fig. Comparison of REE-IC and REE-Harris-Benedict in patients with BMI > 30 kg/m2. S12 Fig. Comparison of REE-IC and REE-Mifflin in patients with BMI > 30 kg/m2. S13 Fig. Comparison of REE_IC and REE-US in patients with sepsis. S14 Fig. Comparison of REE-IC and REE-Penn State in patients with sepsis. S15 Fig. Comparison of REE-IC and REE-Harris-Benedict in patients with sepsis. S16 Fig. Comparison of REE-IC and REE-Mifflin in patients with sepsis. S17 Fig. Comparison of REE-IC and REE-US in patients in the early stage. S18 Fig. Comparison of REE-IC and REE-Penn State in patients in the early stage. S19 Fig. Comparison of REE-IC and REE-Harris-Benedict in patients in the early stage. S20 Fig. Comparison of REE-IC and REE-Mifflin in patients in the early stage. S21 Fig. Comparison of REE-IC and REE-US in patients in middle and late stage. S22 Fig. Comparison of REE-IC and REE-Penn State in patients in middle and late stage. S23 Fig. Comparison of REE-IC and REE-Harris-Benedict in patients in middle and late stage. S24 Fig. Comparison of REE-IC and REE-Mifflin in patients in middle and late stage.
(DOCX)

## Abbreviations

REE: Resting energy expenditure
IC: Indirect calorimetry
CO: cardiac output
MLT: muscle layer thickness
