# Supplementary material for: Accuracy of bedside ultrasound for predicting resting energy expenditure in critically ill patients: A feasibility study
Source: PLoS One. 2025 Jun 18;20(6):e0325751. doi: 10.1371/journal.pone.0325751 (PMC12176121; doi:10.1371/journal.pone.0325751)
Supplement: S1 File — S1 Table. Sedatives and analgesics use during REE-IC monitoring. S2 Table. Sedatives and analgesics use during REE-US monitoring. S3 Table. Oxygen therapy parameters and other parameters of patients before REE monitoring. S4 Table. Correlation coefficients between different formulas and REE-IC in sepsis. S1 Fig. Comparison of REE-IC and REE-US in patients with BMI < 20 kg/m2. S2 Fig. Comparison of REE-IC and REE-Penn State University in patients with BMI < 20 kg/m2. S3 Fig. Comparison of REE-IC and REE-Harris-Benedict in patients with BMI < 20 kg/m2. S4 Fig. Comparison of REE-IC and REE-Mifflin in patients with BMI < 20 kg/m2. S5 Fig. Comparison of REE-IC and REE-US in patients with BMI 20–30 kg/m2. S6 Fig. Comparison of REE-IC and REE-Penn State in patients with BMI 20–30 kg/m2. S7 Fig. Comparison of REE-IC and REE-Harris-Benedict in patients with BMI 20–30 kg/m2. S8 Fig. Comparison of REE-IC and REE-Mifflin in patients with BMI 20–30 kg/m2. S9 Fig. Comparison of REE-IC and REE-US in patients with BMI > 30 kg/m2. S10 Fig. Comparison of REE-IC and REE-Penn State in patients with BMI > 30 kg/m2. S11 Fig. Comparison of REE-IC and REE-Harris-Benedict in patients with BMI > 30 kg/m2. S12 Fig. Comparison of REE-IC and REE-Mifflin in patients with BMI > 30 kg/m2. S13 Fig. Comparison of REE_IC and REE-US in patients with sepsis. S14 Fig. Comparison of REE-IC and REE-Penn State in patients with sepsis. S15 Fig. Comparison of REE-IC and REE-Harris-Benedict in patients with sepsis. S16 Fig. Comparison of REE-IC and REE-Mifflin in patients with sepsis. S17 Fig. Comparison of REE-IC and REE-US in patients in the early stage. S18 Fig. Comparison of REE-IC and REE-Penn State in patients in the early stage. S19 Fig. Comparison of REE-IC and REE-Harris-Benedict in patients in the early stage. S20 Fig. Comparison of REE-IC and REE-Mifflin in patients in the early stage. S21 Fig. Comparison of REE-IC and REE-US in patients in middle and late stage. S22 Fig. Comparison of REE-IC and R [file pone.0325751.s001.docx]

Clinical feasibility of predicting resting energy expenditure in critically ill patients using ultrasound

Ming Gao, MD^1^, Li Tan, MD^1^, Ying-Li Zhou, MD^1^, Wei Peng, MD^1^, Yuan Xu, MD^1^, Hua Zhou, MD^1^, Arthur R. H. van Zanten, MD^2#^, Yan Zhu, MD^1#^

# Corresponding author:

**Yan Zhu**, **M.D.** Department of Critical Care Medicine, Beijing Tsinghua Changgung Hospital, School of Clinical Medicine, Tsinghua University, Beijing 102218, China. Email: zya02226@btch.edu.cn.

**Arthur Raymond Hubert van Zanten, MD, Ph.D.** Department of Intensive Care, Gelderse Vallei Hospital, Willy Brandtlaan 10, 6716 RP Ede, The Netherlands; Division of Human Nutrition and Health, Wageningen University & Research, Helix (Building 124), Stippeneng 4, 6708 WE Wageningen, The Netherlands. Tel: +31 318 434115; fax: +31318434116; e-mail: [zantena@zgv.nl](mailto:zantena@zgv.nl) or arthur.vanzanten@wur.nl

Appendix files

Appendix 1 Method of Ultrasound Evaluation………………… ……………………………..2

Appendix 2 Sedatives and analgesics use during REE-IC and REE-US monitoring………………………………………………………………………………………4

Appendix 3 Table S1 Oxygen therapy parameters and other parameters of patients before REE monitoring………………………………………………………………………………………..6

Appendix 4 Table S2 Correlation coefficients between different formulas and REE-IC in sepsis……………………………………………………………………………………………7

Appendix 5 Fig. S1-S24 Bland–Altman analysis of REE-IC and other equations in different types of population……………………………………………………………………………………8

Appendix 1

Method of Ultrasound Evaluation

1. Evaluation of Muscle Thickness Using Echocardiography
2. The patient was supine, with arms and legs extended and muscles relaxed.
3. The ultrasound was performed using a Phillips CX50 device in two-dimensional mode with a linear transducer to obtain images of surface structures in cross-section in B-mode. We measured the muscle layer thickness (MLT) using a noncompression technique with minimal pressure on the skin.
4. For the arms, we marked indelible points midway between the tip of the acromion and the tip of the olecranon (with the patient's elbow extended and the forearm supinated) of both arms, and we measured the MLT on the arm over the biceps at that point.
5. For the thighs, we marked indelible points of the midpoint between the anterior superior iliac spine and the upper pole of the patella of both legs. We measured the MLT at that point.
6. Muscle ultrasound examinations were performed twice at each anatomic point, and the mean values were calculated after each examination was performed bilaterally.
7. Evaluation of CO Using Echocardiography
8. Left lying position

2）In the early stage of cardiac contraction, the inner diameter of the aortic valve root was measured at the long axis section of the left ventricle near the sternum by transthoracic echocardiography, which is left ventricular outflow tract(LVOT) diameter (Fig. A).

3）Calculation of LVOT Area, Area = (LVOT diameter /2)^2^• π

4）The left ventricular outflow tract velocity time integral (VTI) was measured at the apical 5-chamber cardiac section after the pulse Doppler sampling volume was placed 0.5cm below the aortic valve (Fig. B).

5）Calculation of stroke volume (SV), SV = Area • VTI；

6）Calculation of cardiac output (CO), CO = SV • HR

The calculation above is based on the data package in the ultrasound machine.


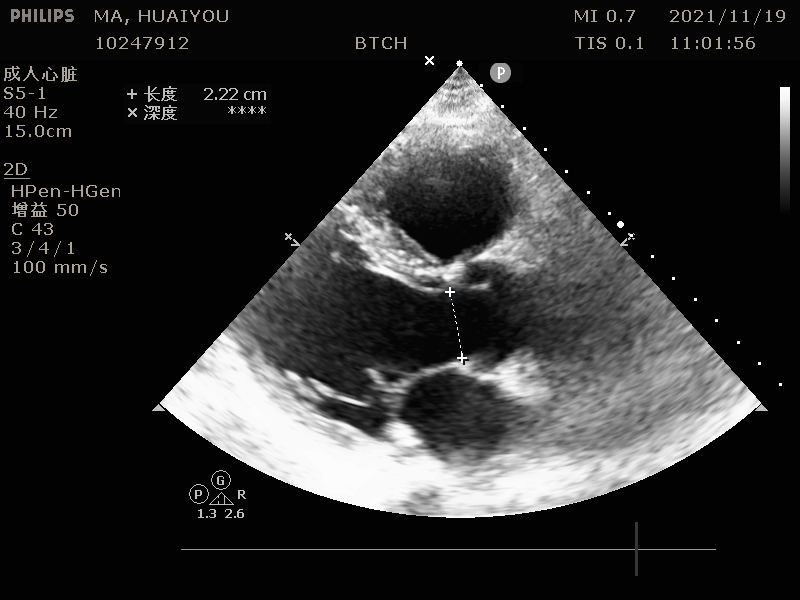


Fig. A. Measurement of the components of CO by US: LVOT diameter.


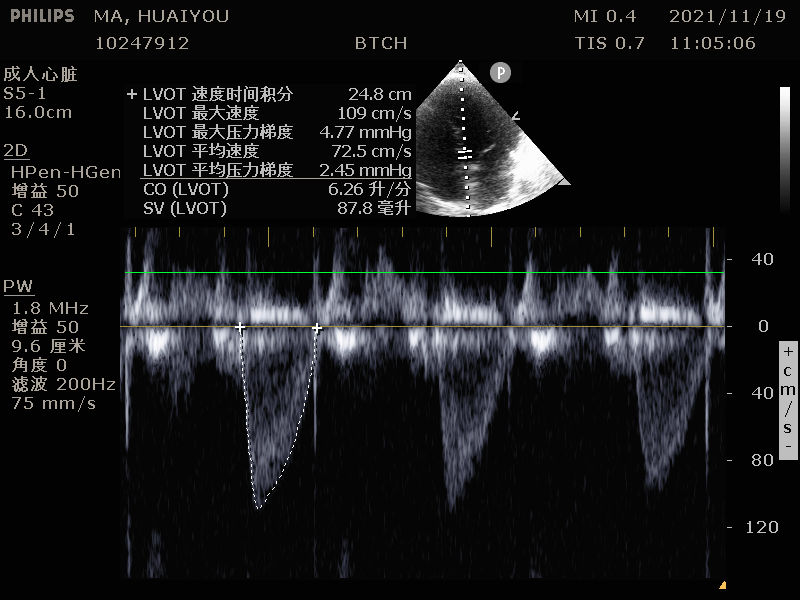


Fig. B. Measurement of the components of CO by US: VTI.

Appendix 2 Sedative use during REE-IC and REE-US monitoring

Tab S1: Sedatives and analgesics use during REE-IC monitoring

| Variable | Total measurements  (n = 124) |
| --- | --- |
| No use of sedatives（n, %） | 66（53.2） |
| Use of sedatives（n, %）* | 58（46.8） |
| Midazolam（n, %）# | 25（43.1） |
| Dose of midazolam（median, IQR, mg/kg/h） | 0.065 (0.041) |
| Propofol（n, %）# | 36（62.1） |
| Dose of propofol（median, IQR, mg/kg/h） | 0.67 (0.35) |
| Dexmedetomidine（n, %）# | 23（39.7） |
| Dose of Dexmedetomidine（mean±SD, μg/kg/h） | 0.35±0.11 |
| RASSscores（n=58）(median, IQR) | -2 (-1) |

***** More than one sedative was used for some patients

# The proportion of a certain drug use is times of the drug use/the total number of sedatives use, e.g., the proportion of using midazolam is 25/58 IQR: interquartile range

Tab S2: Sedatives and analgesics use during REE-US monitoring

| Variable | Total measurements  (n = 124) |
| --- | --- |
| No use of sedatives | 65（52.4） |
| Use of sedatives * | 59（47.6） |
| Midazolam（n, %）# | 29（49.2） |
| Dose of midazolam（median, IQR, mg/kg/h） | 0.065 (0.03) |
| Propofol（n, %）# | 37（62.7） |
| Dose of propofol（median, IQR, mg/kg/h） | 0.67 (0.38) |
| Dexmedetomidine（n, %）# | 23（39.0） |
| Dose of Dexmedetomidine（mean±SD, ug/kg/h） | 0.34±0.11 |
| RASS scores（n=59）(median, IQR) | -2 (-1) |

***** More than one sedative was used for some patients # The proportion of a certain drug use is times of the drug use/the total number of sedatives use, e.g., the proportion of using midazolam is 29/59

IQR: interquartile range

Appendix 3

Tab S3： Oxygen therapy parameters and other parameters of patients before REE monitoring

| Variables | **Total** measurements  (n = 124) |
| --- | --- |
| Invasive mechanical ventilation（n=100） |  |
| Ventilation mode |  |
| PSV（n, %） | 79（63.7） |
| PAC（n, %） | 20（16.1） |
| SIMV+PSV（n, %） | 1（0.81） |
| FiO_2_（%）(mean±SD) | 39.5±4.7 |
| Minute Volume（L/min）(mean±SD) | 7.83±2.0 |
| PEEP（cmH_2_O）(median, IQR) | 6（3） |
| Temperature (℃) (median, IQR) | 36.8 (1) |
| Non-invasive ventilation（n=24） |  |
| Oxygen therapy mode |  |
| Muti-flow（n, %） | 20（16.1） |
| NC（n, %） | 2（1.61） |
| HFNC（n, %） | 2 (1.61) |
| FiO_2_（%）(mean±SD) | 40 (36.3,40) |
| Minute Volume（L/min）(mean±SD) | 8.51±2.3 |
| Temperature (℃) (mean±SD) | 36.9±0.6 |

PSV: pressure support ventilation; PAC: pressure assist/control; SIMV: synchronized intermittent mandatory ventilation; FiO_2_: fraction of inspiration O_2_, NC: nasal catheter; HFNC: High Flow Nasal Cannula.

Appendix 4

Tab S4: Correlation coefficients between different formulas and REE-IC in sepsis

|  | Mifflin formula  （kcal/d） | Harris-Benedict formula  （kcal/d） | Penn State University formula  （kcal/d） | Ultrasound (REE-US)  （kcal/d） | ASPEN standard  （kcal/d） | REE-IC  （kcal/d） |
| --- | --- | --- | --- | --- | --- | --- |
| Measurements with sepsis  (n=33) | 1373± 189  r = 0.560, p = 0.001 | 1356 ± 230  r = 0.569, p = 0.001 | 1610 ± 275  r = 0.661, p<0.001 | 1613 ± 234  r = 0.612, p<0.001 | 1612±280  r = 0.395, p = 0.023 | 1623 ± 372 |

Appendix 5

Fig. S1 Comparison of REE-IC and REE-US in patients with BMI＜20kg/m^2^.

REE indicates resting energy expenditure using indirect calorimetry;

REE-US indicates resting energy expenditure using ultrasound.

Fig. S2 Comparison of REE-IC and REE-Penn State University in patients with BMI＜20kg/m^2^.

REE indicates resting energy expenditure using indirect calorimetry;

REE-Penn State indicates resting energy expenditure using the Penn State University formula.

Fig. S3 Comparison of REE-IC and REE-Harris-Benedict in patients with BMI＜20kg/m^2^.

.

REE indicates resting energy expenditure using indirect calorimetry;

REE-Harris-Benedict indicates resting energy expenditure using the Harris-Benedict formula.

Fig. S4 Comparison of REE-IC and REE-Mifflin in patients with BMI＜20kg/m^2^.

REE indicates resting energy expenditure using indirect calorimetry;

REE-Mifflin indicates resting energy expenditure using the Mifflin formula.

Fig. S5 Comparison of REE-IC and REE-US in patients with BMI 20-30kg/m^2^.

REE indicates resting energy expenditure using indirect calorimetry;

REE-US indicates resting energy expenditure using ultrasound.

Fig. S6 Comparison of REE-IC and REE-Penn State in patients with BMI 20-30kg/m^2^.

REE indicates resting energy expenditure using indirect calorimetry;

REE-Penn State indicates resting energy expenditure using the Penn State University formula.

Fig. S7 Comparison of REE-IC and REE-Harris-Benedict in patients with BMI 20-30kg/m^2^.

REE indicates resting energy expenditure using indirect calorimetry;

REE-Harris-Benedict indicates resting energy expenditure using the Harris-Benedict formula.

Fig. S8 Comparison of REE-IC and REE-Mifflin in patients with BMI 20-30kg/m^2^.

REE indicates resting energy expenditure using indirect calorimetry;

REE-Harris-Benedict indicates resting energy expenditure using the Mifflin formula.

Fig. S9 Comparison of REE-IC and REE-US in patients with BMI＞30kg/m^2^.

REE indicates resting energy expenditure using indirect calorimetry;

REE-US indicates resting energy expenditure using ultrasound.

Fig. S10 Comparison of REE-IC and REE-Penn State in patients with BMI＞30kg/m^2^.

REE indicates resting energy expenditure using indirect calorimetry;

REE-Penn State indicates resting energy expenditure using the Penn State University formula.

Fig. S11 Comparison of REE-IC and REE-Harris-Benedict in patients with BMI＞30kg/m^2^.

REE indicates resting energy expenditure using indirect calorimetry;

REE-Harris-Benedict indicates resting energy expenditure using the Harris-Benedict formula.

Fig. S12 Comparison of REE-IC and REE-Mifflin in patients with BMI＞30kg/m^2^.

REE indicates resting energy expenditure using indirect calorimetry;

REE-Mifflin indicates resting energy expenditure using the Mifflin formula.

Fig. S13 Comparison of REE_IC and REE-US in patients with sepsis.

REE indicates resting energy expenditure using indirect calorimetry;

REE-US indicates resting energy expenditure using ultrasound.

Fig. S14 Comparison of REE-IC and REE-Penn State in patients with sepsis.

REE indicates resting energy expenditure using indirect calorimetry;

REE-Penn State indicates resting energy expenditure using the Penn State University formula.

Fig. S15 Comparison of REE-IC and REE-Harris-Benedict in patients with sepsis.

REE indicates resting energy expenditure using indirect calorimetry;

REE-Harris-Benedict indicates resting energy expenditure using the Harris-Benedict formula.

Fig. S16 Comparison of REE-IC and REE-Mifflin in patients with sepsis.

REE indicates resting energy expenditure using indirect calorimetry;

REE-Mifflin indicates resting energy expenditure using the Mifflin formula.

Fig. S17 Comparison of REE-IC and REE-US in patients in the early stage.

REE indicates resting energy expenditure using indirect calorimetry;

REE-US indicates resting energy expenditure using ultrasound.

Fig. S18 Comparison of REE-IC and REE-Penn State in patients in the early stage.

REE indicates resting energy expenditure using indirect calorimetry;

REE-Penn State indicates resting energy expenditure using the Penn State University formula.

Fig. S19 Comparison of REE-IC and REE-Harris-Benedict in patients in the early stage.

REE indicates resting energy expenditure using indirect calorimetry;

REE-Harris-Benedict indicates resting energy expenditure using the Harris-Benedict formula.

Fig. S20 Comparison of REE-IC and REE-Mifflin in patients in the early stage.

REE indicates resting energy expenditure using indirect calorimetry;

REE-Mifflin indicates resting energy expenditure using the Mifflin formula.

Fig. S21 Comparison of REE-IC and REE-US in patients in middle and late stage.

REE indicates resting energy expenditure using indirect calorimetry;

REE-US indicates resting energy expenditure using ultrasound.

Fig. S22 Comparison of REE-IC and REE-Penn State in patients in middle and late stage.

REE indicates resting energy expenditure using indirect calorimetry;

REE-Penn State indicates resting energy expenditure using the Penn State University formula.

Fig. S23 Comparison of REE-IC and REE-Harris-Benedict in patients in middle and late stage.

REE indicates resting energy expenditure using indirect calorimetry;

REE-Harris-Benedict indicates resting energy expenditure using the Harris-Benedict formula.

Fig. S24 Comparison of REE-IC and REE-Mifflin in patients in middle and late stage.

REE indicates resting energy expenditure using indirect calorimetry;

REE-Mifflin indicates resting energy expenditure using the Mifflin formula.
